# Supplementary figures and images for: Immunometabolic signatures predict risk of progression to sepsis in COVID-19
Source: PLoS One. 2021 Aug 30;16(8):e0256784. doi: 10.1371/journal.pone.0256784 (PMC8405033; doi:10.1371/journal.pone.0256784)

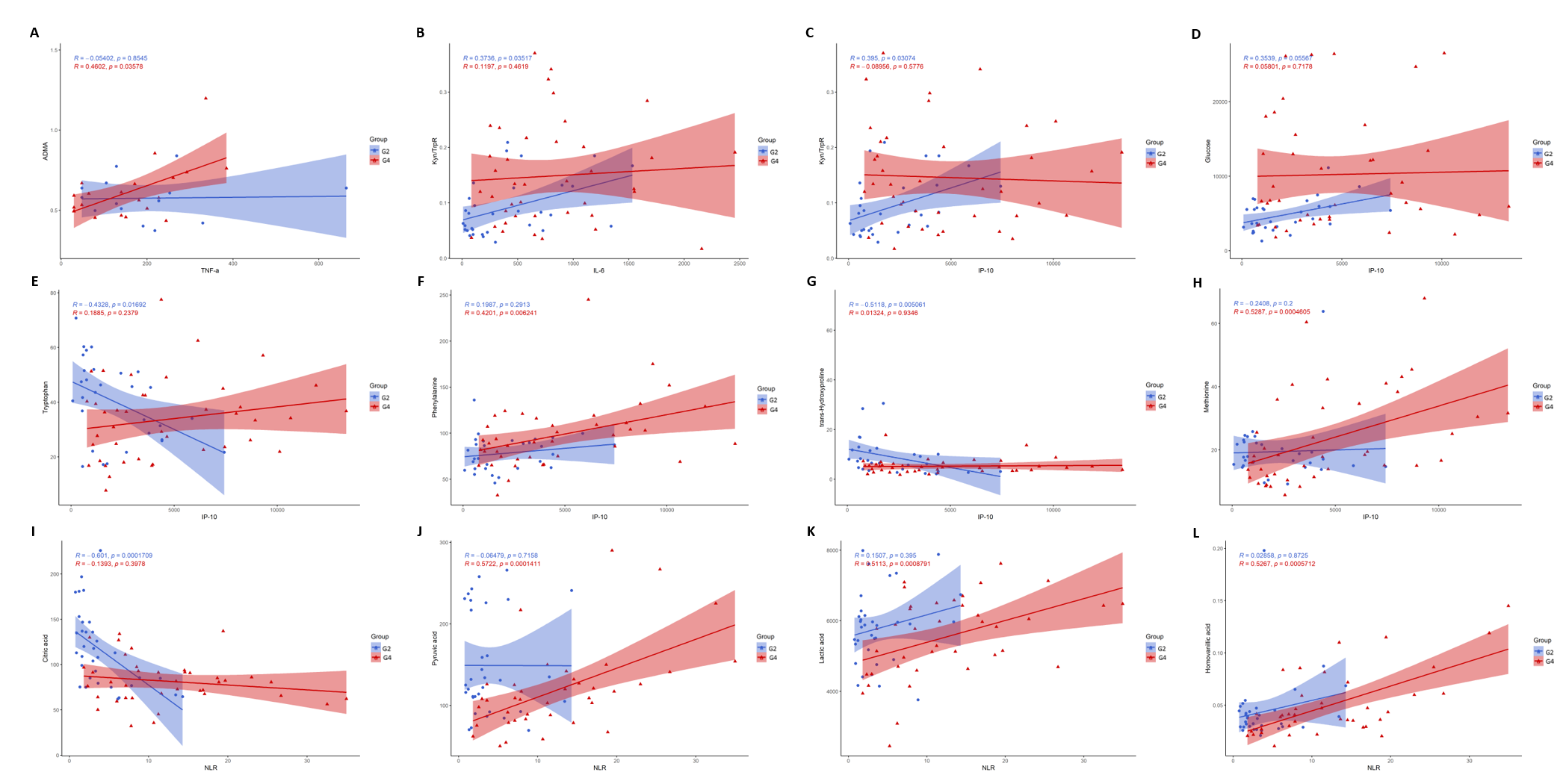

Supplement: S1 Fig — (A) ADMA vs. TNF-α (B) kynurenine: tryptophan ratio vs. IL-6 (C) kynurenine: tryptophan ratio vs. IP-10 (D) glucose vs. IP-10 (E) tryptophan vs. IP-10 (F) phenylalanine vs. IP-10 (G) trans- hydroxyproline vs. IP-10 (H) methionine vs. IP-10 (I) citric acid vs. NLR (J) pyruvic acid vs. NLR (K) lactic acid vs. NLR (L) homovallinic acid vs. NLR. Spearman correlations with p-values are shown for each correlation. (TIF) [file pone.0256784.s001.tif]

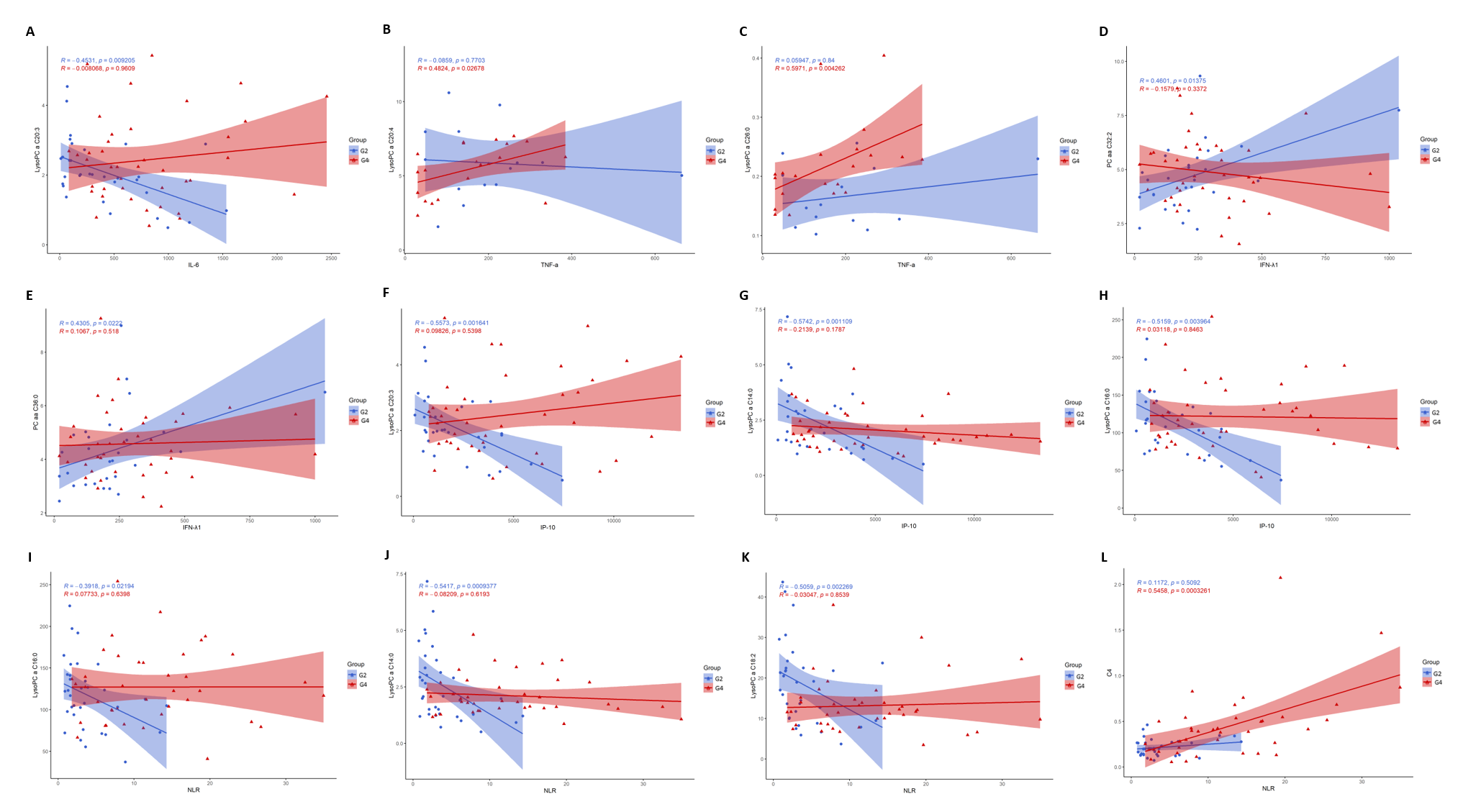

Supplement: S2 Fig — (A) LysoPC a 20:3 vs. IL-6 (B) LysoPC a 20:4 vs. TNF-α (C) LysoPC a 26:0 vs. TNF-α (D) PC aa 32:2 vs. IFN λ1 (E) PC aa 36:0 vs. IFN λ1 (F) LysoPC a 20:3 vs. IP-10 (G) LysoPC a 14:0 vs. IP-10 (H) LysoPC a 16:0 vs. IP-10 (I) LysoPC a 16:0 vs. NLR (J) LysoPC a 14:0 vs. NLR (K) LysoPC a 18:2 vs. NLR (L) C4 vs. NLR. Spearman correlation coefficients with p-values are shown for each correlation. (TIF) [file pone.0256784.s002.tif]

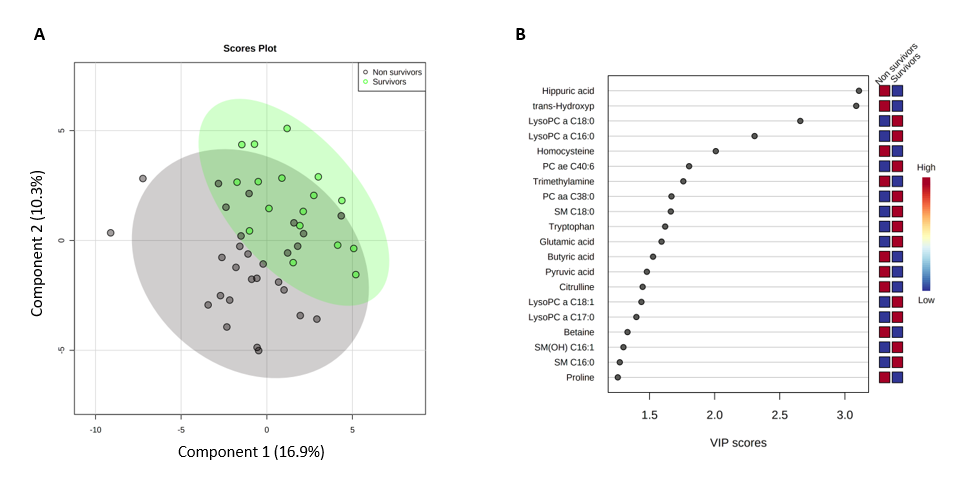

Supplement: S3 Fig — (A) Multivariate analysis from plasma metabolome profile of non-survivors vs. survivors from G4 Score scatter plot based on the PLS-DA models to explain the mortality (black: non-survivors, green: survivors). (B) rank of the top 20 metabolites identified by PLS-DA using VIP score on x- axis. The most discriminating metabolites are shown by descending coefficient scores coefficients. The color boxes indicate increased (red) or decreased (blue) metabolite concentrations. (TIF) [file pone.0256784.s003.tif]

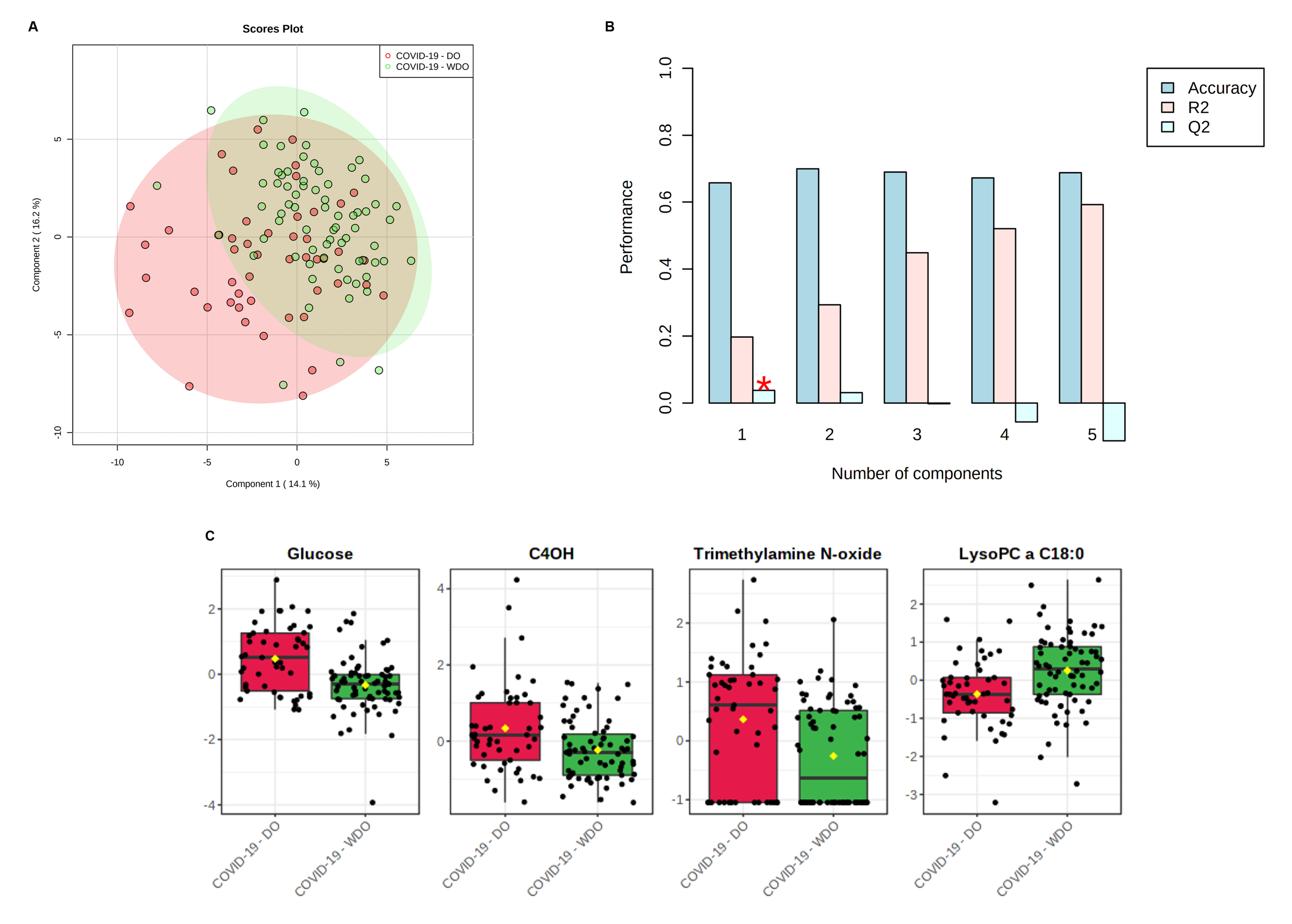

Supplement: S4 Fig — (A) Score scatter plot based on PLS-DA models to explain the diagnosis (COVID-WDO: green, COVID-DO: red). (B) 10-cross validation of PLS-DA. (C) Univariate analysis showing the four significant metabolites. The color boxes indicate whether metabolite concentration was increased (red) or decreased (green). Figures were produced in MetaboAnalyst software v 4.0 (https://www.metaboanalyst.ca/). (TIF) [file pone.0256784.s004.tif]
