# Supplementary material for: Immunometabolic signatures predict risk of progression to sepsis in COVID-19
Source: PLoS One. 2021 Aug 30;16(8):e0256784. doi: 10.1371/journal.pone.0256784 (PMC8405033; doi:10.1371/journal.pone.0256784)
Supplement: S1 Table — (DOCX) [file pone.0256784.s005.docx]

**S1 Table. Clinical and demographic characteristics of survivors and non-survivors from critically ill group of patients.**

| Variables | Survivors Sepsis (N = 17) | Non-survivors Sepsis (N = 28) | P value |
| --- | --- | --- | --- |
| Male sex, n (%) | 11 (64.7) | 18 (64.3) | >0.99 |
| Age, median years, mean ± SD | 52 ± 11 | 57 ± 10 | 0.15 |
| Symptoms to sampling, median days (Q1-Q3) | 5 (2-7) | 8 (5-15) | **0.01** |
| Symptoms antes de admission (Q1-Q3) | 5 (2-7) | 4 (2-7) | 0.7 |
| Comorbidities, n (%) |  |  |  |
| Diabetes | 1 (5.8) | 10 (35.7) | **0.03** |
| Obesity | 3 (17.7) | 13 (46.4) | 0.06 |
| Hypertension | 8 (47) | 13 (46.4) | >0.99 |
| Admission Lab data |  |  |  |
| Platelets (thousands/ ml) | 206.5 ± 90 | 257.5 ± 73.92 | 0.05 |
| Neutrophils (%) | 85 (80.2-91.5) | 86.3 (78.8-91.2) | 0.7 |
| Lymphocytes (%) | 10.3 (5.1-14.8) | 6.7 (4.6-11.3) | 0.2 |
| Neutrophils-Lymphocytes Radio | 11.2 ± 6.4 | 13.4 ± 9.3 | 0.4 |
| Monocytes (%) | 3.2 (2.8-4.7) | 3.4 (2.5-5.7) | 0.8 |
| Cytokine panel, (pg/ml) | | | |
| IL-1β | 270.8 (94.5-460) | 191.4 (118.5-395) | 0.9 |
| IL-6 | 1021 ± 452 | 544.4 ± 283.5 | **0.0004** |
| TNF-α | 177.5 ± 132.7 | 142.5 ± 90 | 0.5 |
| IP-10 | 3963 (2732-8190) | 2672 (1397-6146) | 0.08 |
| IFN-λ1 | 336.1 ± 167 | 232.8 ± 130 | **0.04** |
| IL-8 | 259.6 (185.0-454.2) | 232.6 (130.8-289.7) | 0.5 |
| IL-12p70 | 49.6 (30.5-73.1) | 34.4 (14.8-52) | 0.1 |
| IFN-α2 | 134 (45.2-216.6) | 66.6 (31.6-137.4) | 0.2 |
| IFN-λ2/3 | 143 ± 66.3 | 146.7 ± 76.8 | 0.9 |
| GM-CSF | 42.1 (23.2-95.8) | 78.4 (16.2-158.5) | 0.6 |
| IFN-β | 250 (70-367) | 92.1 (29-236) | 0.08 |
| IL-10 | 359 (247.6-464.2) | 268 (170-342.4) | 0.09 |
| IFN-γ | 205.4 ± 104 | 231.2 ± 150.1 | 0.6 |
