# Supplementary material for: Immunometabolic signatures predict risk of progression to sepsis in COVID-19
Source: PLoS One. 2021 Aug 30;16(8):e0256784. doi: 10.1371/journal.pone.0256784 (PMC8405033; doi:10.1371/journal.pone.0256784)
Supplement: S2 Table — (DOCX) [file pone.0256784.s006.docx]

**S2 Table. Logistics regression based optimal models adjusted for diabetes and obesity**

| **Logistic Regression Model with Selected Compounds:**  logit(P) = log(P / (1 - P)) = 0.306 - 0.683 Obesity - 0.291 Diabetes  The optimal cut-off point for the above equation is 0.45. | | | | | |
| --- | --- | --- | --- | --- | --- |
| **Metabolites and citokynes** | **Estimate** | **Std. Error** | **z value** | **Pr(>\|z\|)** | **Odds** |
| (Intercept) | 0.306 | 0.247 | 1.236 | 0.217 | - |
| Obesity | -0.683 | 0.299 | -2.284 | 0.022 | 0.51 |
| Diabetes | -0.291 | 0.285 | -1.021 | 0.307 | 0.75 |

**G1: PCR-/controls, G4: PCR+ critical/sepsis patients.**

| **Logistic Regression Model with Selected Compounds:**  logit(P) = log(P / (1 - P)) = 2.367 + 1.658 Kynurenine/Tryptophan + 1.836 Phenylalanine - 5.373 LysoPC a C18:2 + 2.685 IL-6 (pg/mL) - 1.045 Obesity + 1.61 Diabetes  The optimal cut-off point for the above equation is 0.52 | | | | | |
| --- | --- | --- | --- | --- | --- |
| **Metabolites/citokynes /comorbidities** | **Estimate** | **Std. Error** | **z value** | **Pr(>\|z\|)** | **Odds** |
| (Intercept) | 2.367 | 1.147 | 2.064 | 0.039 | - |
| Kynurenine/Tryptophan | 1.658 | 0.825 | 2.011 | 0.044 | 5.25 |
| Phenylalanine | 1.836 | 0.831 | 2.21 | 0.027 | 6.27 |
| LysoPC a C18:2 | -5.373 | 2.671 | -2.011 | 0.044 | 0 |
| IL-6 (pg/mL) | 2.685 | 1.303 | 2.06 | 0.039 | 14.66 |
| Obesity | -1.045 | 0.978 | -1.069 | 0.285 | 0.35 |
| Diabetes | 1.61 | 1.202 | 1.339 | 0.181 | 5 |

**G1: PCR-/controls, G4: PCR+ critical/sepsis patients.**

| **Logistic Regression Model with Selected Compounds:**  logit(P) = log(P / (1 - P)) = 0.111 - 0.274 Obesity - 0.336 Diabetes  The optimal cut-off point for the above equation is 0.51 | | | | | |
| --- | --- | --- | --- | --- | --- |
| **Metabolites and citokynes** | **Estimate** | **Std. Error** | **z value** | **Pr(>\|z\|)** | **Odds** |
| (Intercept) | 0.111 | 0.223 | 0.496 | 0.62 | - |
| Obesity | -0.274 | 0.239 | -1.151 | 0.25 | 0.76 |
| Diabetes | -0.336 | 0.252 | -1.335 | 0.182 | 0.71 |

**G2: PCR+/not hospitalized, G4: PCR+ critical/sepsis patients.**

| **Logistic Regression Model with Selected Compounds:**  logit(P) = log(P / (1 - P)) = 0.254 + 0.763 C5 + 1.038 Neutro/Linfo Radio NLR + 4.298 C10:2 + 0.873 IL-6 (pg/mL) - 0.098 Diabetes - 0.164 Obesity  The optimal cut-off point for the above equation is 0.27 | | | | | |
| --- | --- | --- | --- | --- | --- |
| **Metabolites/ citokynes /comorbidities** | **Estimate** | **Std. Error** | **z value** | **Pr(>\|z\|)** | **Odds** |
| (Intercept) | 0.254 | 0.426 | 0.597 | 0.551 | - |
| C5 | 0.763 | 0.493 | 1.548 | 0.122 | 2.15 |
| Neutro/Linfo Radio NLR | 1.038 | 0.436 | 2.378 | 0.017 | 2.82 |
| C10:2 | 4.298 | 1.153 | 3.728 | < 0.001 | 73.54 |
| IL-6 (pg/mL) | 0.873 | 0.463 | 1.888 | 0.059 | 2.39 |
| Diabetes | -0.098 | 0.522 | -0.188 | 0.851 | 0.91 |
| Obesity | -0.164 | 0.587 | -0.278 | 0.781 | 0.85 |

**G2: PCR+/not hospitalized, G4: PCR+ critical/sepsis patients.**

| **Logistic Regression Model with Selected Compounds:**  logit(P) = log(P / (1 - P)) = 0.271 - 0.66 Diabetes + 0.422 Obesity  The optimal cut-off point for the above equation is 0.59. | | | | | |
| --- | --- | --- | --- | --- | --- |
| **Metabolites and citokynes** | **Estimate** | **Std. Error** | **z value** | **Pr(>\|z\|)** | **Odds** |
| (Intercept) | 0.271 | 0.238 | 1.139 | 0.255 | - |
| Diabetes | -0.66 | 0.253 | -2.609 | 0.009 | 0.52 |
| Obesity | 0.422 | 0.257 | 1.643 | 0.1 | 1.53 |

**G3: PCR+ moderated/severe patients, G4: PCR+ critical/sepsis patients both hospitalized**

| **Logistic Regression Model with Selected Compounds:**  logit(P) = log(P / (1 - P)) = 0.42 + 0.556 Kynurenine/Tryptophan + 0.751 LysoPC a C28:1 - 0.86 Citric acid + 0.38 Neutro/Linfo Radio NLR - 0.553 Diabetes + 0.324 Obesity  The optimal cut-off point for the above equation is 0.48 | | | | | |
| --- | --- | --- | --- | --- | --- |
| **Metabolites and citokynes** | **Estimate** | **Std. Error** | **z value** | **Pr(>\|z\|)** | **Odds** |
| (Intercept) | 0.42 | 0.288 | 1.458 | 0.145 | - |
| Kynurenine/Tryptophan | 0.556 | 0.284 | 1.955 | 0.051 | 1.74 |
| LysoPC a C28:1 | 0.751 | 0.295 | 2.544 | 0.011 | 2.12 |
| Citric acid | -0.86 | 0.332 | -2.587 | 0.01 | 0.42 |
| Neutro/Linfo Radio NLR | 0.38 | 0.292 | 1.303 | 0.193 | 1.46 |
| Diabetes | -0.553 | 0.3 | -1.843 | 0.065 | 0.58 |
| Obesity | 0.324 | 0.3 | 1.083 | 0.279 | 1.38 |

**G3: PCR+ moderated/severe patients, G4: PCR+ critical/sepsis patients both hospitalized**
